# *Schizachyrium scoparium* (C_4_) better tolerates drought than *Andropogon gerardii* (C_4_) via constant CO_2_ supply for photosynthesis during water stress

**DOI:** 10.1093/aobpla/plae012

**Published:** 2024-03-08

**Authors:** Alina Dekirmenjian, Diego Montano, Michelle L Budny, Nathan P Lemoine

**Affiliations:** Department of Biological Sciences, Marquette University, 1428 W Clybourn St, Milwaukee, WI 53233USA; Department of Biological Sciences, Marquette University, 1428 W Clybourn St, Milwaukee, WI 53233USA; Department of Biological Sciences, Marquette University, 1428 W Clybourn St, Milwaukee, WI 53233USA; Department of Biological Sciences, Marquette University, 1428 W Clybourn St, Milwaukee, WI 53233USA; Department of Zoology, Milwaukee Public Museum, 800 W Wells St, Milwaukee, WI 53201USA

**Keywords:** Drought, ecophysiology, grasslands, prairies, water use

## Abstract

Abstract. Climate change is dramatically altering global precipitation patterns across terrestrial ecosystems, making it critically important that we understand both how and why plant species vary in their drought sensitivities. *Andropogon gerardii* and *Schizachyrium scoparium*, both C_4_ grasses, provide a model system for understanding the physiological mechanisms that determine how species of a single functional type can differ in drought responses, an issue remains a critical gap in our ability to model and predict the impacts of drought on grassland ecosystems. Despite its greater lability of foliar water content, previous experiments have demonstrated that *S. scoparium* maintains higher photosynthetic capacity during droughts. It is therefore likely that the ability of *S. scoparium* to withstand drought instead derives from a greater metabolic resistance to drought. Here, we tested the following hypotheses: (H1) *A. gerardii* is more vulnerable to drought than *S. scoparium* at both the population and organismal levels, (H2) *A. gerardii* is less stomatally flexible than *S. scoparium*, and (H3) *A. gerardii* is more metabolically limited than *S. scoparium*. Our results indicate that it is actually stomatal limitations of CO_2_ supply that limit *A. gerardii* photosynthesis during drought. *Schizachyrium scoparium* was more drought-resistant than *A. gerardii* based on long-term field data, organismal biomass production and physiological gas exchange measurements. While both *S. scoparium* and *A. gerardii* avoided metabolic limitation of photosynthesis, CO_2_ supply of *A. gerardii* was greatly reduced during late-stage drought stress. That two common, co-occurring C_4_ species possess such different responses to drought highlights the physiological variability inherent within plant functional groups and underscores the need for more studies of C_4_ drought tolerance.

## Teaser

Despite their outward similarity, co-existing grasses that occupy the same physiological niche can exhibit very different responses to drought. In North America, there are two co-occurring, physiologically similar grass species, *Andropogon gerardii* and *Schizachyrium scoparium* , that exhibit quite different drought tolerances, but no study has yet identified the physiological reasons behind their different drought responses. Our study illustrated that two common, co-occurring C4 species possess very different responses to drought, highlighting the physiological variability inherent within plant functional groups.

## Introduction

Climate change is dramatically altering global precipitation patterns across terrestrial ecosystems (Sheffield and Wood 2008; IPCC 2014), making it critically important that we understand both how and why plant species vary in their drought sensitivities (Wilcox *et al.* 2020). In ecological studies, drought responses are most often compared among broad categories of plants, such as life history strategies (i.e. grasses and forbs, (Lemoine and Smith 2019)) or photosynthetic pathways (i.e. C_3_ and C_4_ plants, (Lemoine *et al.* 2018)). However, these categories may be too broad given that co-occurring species within a single photosynthetic group can exhibit highly divergent responses to water stress (Ripley *et al.* 2010; Taylor *et al.* 2012, 2014; Wilcox *et al.* 2016, 2020). For example, the North American Great Plains cover roughly 33% of the USA and are often composed of co-occurring C_4_ grasses; yet different locations in the Great Plains can exhibit vastly different responses to drought, likely because different grasses possess different responses to water limitation (Lemoine 2021). Drought reduces production in western dry prairies, dominated by shortgrass C_4_ species, more than in eastern mesic prairies that are comprised of tallgrass C_4_ species (Carroll *et al.* 2021). Even within a single prairie, C_4_ grasses differ in their sensitivity to water stress. Irrigation in a mesic tallgrass prairie caused a shift in community composition from *Andropogon gerardii* to *Panicum virgatum*, though both are C_4_ grasses (Wilcox *et al.* 2016). Clearly, species within a functional group possess markedly different drought responses both among and within communities, and this issue remains a critical gap in our ability to model and predict the impacts of drought on grassland ecosystems.

Two co-existing C_4_ NADP-ME grasses, *A. gerardii* and *Schizachyrium scoparium*, provide a model system for understanding how species within the same functional group can differ in drought responses. During the extreme drought years of the 1930s in North America (i.e. the ‘Dust Bowl’), *S. scoparium* replaced *A. gerardii* as the dominant grass throughout much of the Great Plains (Weaver and Albertson 1943). Recent long-term studies confirmed that aboveground production of *A. gerardii* is more sensitive to interannual rainfall variation than that of *S. scoparium* (La Pierre *et al.* 2011). Indeed, *S. scoparium* flower production declines by only 5–20% during drought years, whereas flower production of *A. gerardii* drops by 25–50% during those same years (Lemoine *et al.* 2017). Controlled experiments have confirmed the greater sensitivity of *A. gerardii* to drought, showing that total plant height was reduced earlier and more severely for *A. gerardii* during drought than for *S. scoparium* (Maricle *et al.* 2015). Interestingly, the different drought sensitivities of *S. scoparium* and *A. gerardii* likely arise because these two C_4_ species possess different stomatal and metabolic responses to water stress.

The water relations of *S. scoparium* and *A. gerardii* suggest that *S. scoparium* should, in fact, be more impacted by drought than *A. gerardii*. Over the course of an abnormally dry growing season, *A. gerardii* maintained constant water potentials between 0 and −1.5 MPa despite soil volumetric water content declining by 50% (Hake *et al.* 1984). During the same drydown period, *S. scoparium* water potentials dropped from 0 to −4.5 MPa (Hake *et al.* 1984). To offset this water loss, *S. scoparium* tends to reduce stomatal conductance to water vapour (*g*_sw_) more severely than *A. gerardii* (Heckathorn *et al.* 1997; Maricle and Adler 2011). Yet despite the potential for stomatal diffusion limitation of photosynthesis, *S. scoparium* maintains higher photosynthetic capacity than *A. gerardii* during droughts (Heckathorn *et al.* 1997; Maricle and Adler 2011; Maricle *et al.* 2015). Since *S. scoparium* can maintain photosynthesis under conditions of low CO_2_ diffusion, it is likely metabolically superior than *A. gerardii*, which loses photosynthetic capacity despite keeping stomata relatively open. It is therefore likely that *A. gerardii* drought sensitivity derives from an inability to maintain metabolic function during periods of water stress.

Metabolic limitations, such as those arising from reduced electron transport and RuBP regeneration, are the primary mechanism by which drought reduces net carbon assimilation (*A*) in many C_4_ grasses (Signarbieux and Feller 2011; Reed and Loik 2016). In contrast to C_3_ species, the relationship between *A* and *g*_sw_ in C_4_ species becomes progressively uncoupled as drought intensifies, signifying that rates of photosynthesis become less limited by CO_2_ supply, and therefore more limited by metabolic processes, during drought (Ripley *et al.* 2010). There are a number of possible factors that limit C_4_ photosynthesis during drought, including decreased activity of both C_3_ and C_4_ enzymes (Rubisco, PEPC, NADP-ME and PPDK) and ATP generation, decreased nitrate assimilation, lower chlorophyll content and reduced photorespiration resulting in a suppression of the electron transport chain (reviewed by Ghannoum 2009; Lawlor and Tezara 2009). Regardless of the precise mechanism, it is clear that the degree of metabolic limitation varies even among C_4_ species. For example, water deficiency induced relative metabolic limitations of photosynthesis ranging from 50% to 80% among three African C_4_ grasses (Ripley *et al.* 2010). In a comparison of six other C_4_ species, Cano *et al.* (2019) showed that species differed considerably in whether photosynthesis was limited by stomatal conductance, boundary layer conductance, or metabolic regeneration of Rubisco. Therefore, it is possible that the different drought responses of *S. scoparium* and *A. gerardii* arise from different degrees of metabolic limitation imposed by water stress, even within C_4_ species.

Here, we coupled long-term observational time series with a physiological lab experiment to understand the mechanisms by which two co-occurring C_4_ grasses differ in drought sensitivity. Based on previous studies, we postulated that photosynthesis of *A. gerardii* would be more drought-limited despite the greater reduction in stomatal conductance of *S. scoparium*. This would occur because *S. scoparium* can metabolically maintain photosynthesis under limited CO_2_ diffusion, compared to the greater metabolic limitation of photosynthesis in *A. gerardii*. Specifically, we tested the following hypotheses:

**H1 *A. gerardii* is more vulnerable to drought than *S. scoparium* at both the population and organismal levels.** We tested this hypothesis with both impulse-response analysis of decadal time series of aboveground biomass and physiological lab experiments. If true, we expected that the time series analysis would show that production of *A. gerardii* declines more during extreme drought than *S. scoparium*. This field-based pattern should be matched by observations that drought reduces growth rate, productivity, photosynthesis and leaf chlorophyll content of *A. gerardii* more than *S. scoparium* in a physiological lab experiment.

**H2 *S. scoparium* reduces stomatal conductance more than *A. gerardii* during drought.** We expected that *S. scoparium* will exhibit a larger degree of foliar water loss under drought and would respond by limiting *g*_sw_ to restrict further water loss (Maricle and Adler 2011). However, the internal CO_2_ concentrating mechanism of the C_4_ cycle should prevent drastic reductions in intercellular CO_2_ concentrations (*Ci*) in either species.

**H3 *A. gerardii* is more metabolically-limited than *S. scoparium*.** If true, we expected to see that the relative degree of stomatal limitation for *S. scoparium* to be higher than that of *A. gerardi*, while the relative degree of metabolic limitation will be higher for *A. gerardii* than for *S. scoparium*. This pattern would result in a significant trade-off between stomatal limitation and metabolic limitation of photosynthesis during drought.

## Methods

### Time series methods and analysis

We tested our first hypothesis that *A. gerardii* is more susceptible than *S. scoparium* to interannual rainfall variability by using publicly available, long-term data provided by the Konza Prairie LTER. While Konza does not collect species-specific aboveground biomass each year, the site does report yearly flowering stalk production of *A. gerardii* and *S. scoparium* (dataset PRE022). Since flowering stalk production can comprise greater than 70% of aboveground biomass (Knapp and Hulbert 1986), fluctuations in flowering stalk biomass likely reflect the total drought sensitivity of grass species at Konza (Knapp and Hulbert 1986; La Pierre *et al.* 2011). Flowering stalk biomass for the C_4_ rhizomotous grasses has been recorded every year since 1983 along eight permanently located 50 m transects (*n *= 4 transects per upland and lowland site). Along each transect, six 0.5 × 0.5 m quadrats were sampled in October each year. All flowering stems of the target grasses were counted and harvested for biomass.

Data processing proceeded by first averaging biomass first across quadrats within transects and then across transects within years. The result was average annual flowering stalk production per 0.25 m^−2^ for both species. These data were then combined with daily precipitation data (Konza LTER dataset AWE012), wherein rainfall was summed for the growing season months (April—September) for each year to produce an annual estimate of water availability. Importantly, we focussed only on an unburned watershed (001D) in upland (Florence) soils, which are known to be more strongly water-limited than downslope Tully soils in the Konza Prairie ecosystem (Lemoine *et al.* 2017).

After data processing, we followed the procedure described by Lemoine (2021) to calculate resistance to an extreme 2*σ* drought. First, both precipitation and flowering stalk biomass were temporally detrended, and the detrended values then standardized to *N*(0, 1). Standardized data were analysed using AR(0), AR(1) and AR(2) models with precipitation as an exogenous variable. The best model was chosen via BIC, and then resistance to a 2*σ* drought determined using an impulse response function (Lemoine 2021). The resistance value describes the relative suppression of flowering stalk production to an extreme drought, with more negative numbers indicating a greater reduction in biomass. Since temporal dynamics of both species were best described by AR(0) models (see Results, see Supporting Information—Table S1), resistance to a 2*σ* drought was calculated as

resistance = *β*(−2)

where *β* is the coefficient of the relationship between annual precipitation and flowering stalk production and −2 represents a 2*σ* reduction in rainfall (Lemoine 2021, see Supporting Information—Fig. S1). This method enabled us to assess how a drought of the same severity affects flowering stalk production in both species, while controlling for potential lag effects and other temporal trends.

### Growth chamber experiment

To evaluate the physiological mechanisms that underpinned our observational study, we conducted a growth chamber experiment using both *A. gerardii* and *S. scoparium*. We conducted our experiment in a Conviron GEN1000 environmental chamber set to a 15-9 photoperiod, with temperature set at a constant 24 °C and relative humidity set to 85% to prevent soil drying.

*Andropogon gerardii* and *S. scoparium* were germinated in separate baking pans filled with potting soil mix on August 15. Seeds were sown randomly on the surface and misted with water daily. On September 13, seedlings were transferred to individual cone-tainers (SC7 cone-tainers from Greenhouse Megastore, one seedling per cone-tainer) filled with potting soil and watered until soil was saturated. On October 7th, we randomly assigned cone-tainers to either control or drought treatments for *A. gerardii* (*n* = 13 per treatment) and *S. scoparium* (*n* = 10 per treatment). Control replicates continued to receive 5 ml of water daily while drought replicates received only 2 ml of water daily.

We tested for drought impacts by measuring leaf relative water content of each species during weeks 4 and 7. We clipped a single leaf from each pot and measured fresh weight using an Ohaus Field Scout scale. Leaves were then rehydrated overnight and reweighed to record fully hydrated weight. We then dried leaves at 60 °C for at least 48 h and recorded dry weight. Relative water content was then calculated as RWC = (fresh weight—dry weight)/(hydrated weight—dry weight).

Each week, we recorded the height of tallest tiller (cm). Mean relative growth rate was calculated for every individual plant as the average of Height_*t*+1_/Heigh_*t*_ across all time points *t* in the experiment. At the end of the experiment, we harvested all above and belowground biomass, dried all biomass at 60 °C for at least 48 h, and weighed all biomass to the nearest 0.001 g.

During weeks 0, 1, 3, 5 and 7, we measured instantaneous leaf gas exchange using an LI-6800 gas exchange system (LI-COR Biosciences). For each individual, we enclosed the newest fully expanded leaf within the instrument leaf chamber. Leaf chamber conditions were set to mimic growth chamber conditions as closely as possible (temperature: 24 °C, relative humidity: 50%, CO_2_: 420 μmol mol^−1^, light: 1500 μmol m^−2^ s^−1^). Flow rate was set to 400 μmol s^−1^. We allowed *A* and *g*_sw_ to reach a steady state for 90–180 s prior to recording each measurement. Because individuals leaves often did not fill the chamber, we measured the width of each leaf using callipers and adjusted the leaf-width parameter to account for different leaf sizes.

During weeks 2 and 4, we measured *A*-*Ci* curves using the dynamic assimilation technique (Saathoff and Welles 2021), where *A* is measured along a continuous CO_2_ concentration ramp. The youngest fully expanded leaf was clamped in the chamber and acclimated at the starting CO_2_ concentration (10 μmol mol^−1^) for 2 min. After the acclimation period, the LI6800 recorded *A* every second as CO_2_ concentration continuously increased from 10 μmol mol^−1^ to 1500 μmol mol^−1^ at a rate of 200 μmol mol^−1^ min^−1^. The DA technique was tested prior to use and found to yield identically shaped curves as the steady-state *A*-*Ci* method (see Supporting Information—Fig. S2). Note that the DA technique has not been systematically validated for C_4_ species, and thus our results might differ from earlier studies using the steady-state approach.

### Growth chamber data analysis

We first calculated relative stomatal and metabolic limitation using the *A*-*Ci* curves. For each curve, we fit an asymptotic monomolecular function of the form:

A = a – (a - b)*e ^*-cCi*^

We quantified relative stomatal limitation from each curve following Ripley *et al.* (2007) and Ripley *et al.* (2010). Briefly,

RSL = [(*A*_40-drought_ – *A*_obs-drought_)/*A*_obs-control_] * 100

RML = [(*A*_obs-control_ – *A*_40-drought_)/*A*_obs-control_]*100

where RSL is relative stomatal limitation, RML is relative metabolic limitation, *A*_40-drought_ is photosynthetic rate at an atmospheric CO_2_ concentration of 40 Pa with no stomatal limitation for Drought plants, *A*_obs-drought_ is photosynthetic rate with prevailing stomatal limitation for drought plants, and *A*_obs-control_ are observed photosynthetic rates for control plants with stomatal limitation. For both RSL and RML, the average *A*_obs-control_ of each species was used (Ripley *et al.* 2007). These calculations are graphically depicted in Fig. 5.

We analysed all measurements using either a one-way or two-way ANOVA, with week of measurement as the second factor where appropriate. A significant interaction indicates that the effect of drought varies with timing of the measurement. All analyses were conducted within a hierarchical Bayesian framework to place weakly informative priors on hyperparameters that constrain estimates of effect sizes when sample sizes are small (Lemoine *et al.* 2016; Lemoine 2019). Each analysis was a linear model of the form:

**y** ~ *N*(**Xb**, *σ*^2^)

where **X** is the design matrix for each analysis and **b** is the vector of coefficients. Coefficients were modelled hierarchically to allow for partial pooling that constrains effect sizes (Lemoine 2019):

**b** ~ *N*(0, *σ*_b_^2^)

The parameter *σ*_b_^2^ was given a weakly informative *Gamma*(2, 0.1) prior, which states that coefficients should be small unless strongly supported by the data. This prior effectively biases the analysis against finding significant effects from small sample sizes unless the effect sizes are vary large and/or consistent among replicates, making the test more conservative than a traditional ANOVA. Although posterior probabilities are best judged as providing a continuous estimate of evidence for an effect, we assessed the ‘significance’ of the results by calculating the probability that a parameter was either positive or negative, wherein Pr > 0.90 indicates a moderately significant effect and Pr > 0.95 indicates a statistically significant effect (Rode *et al.* 2017; Lemoine *et al.* 2018; Lemoine and Budny 2022). All models were via the STAN programming language accessed from Python via the *cmdstanpy* module.

All raw data, cleaned data, Python scripts and figures are available on Figshare at 10.6084/m9.figshare.24319147.

## Results

*H1: A. gerardii was more vulnerable to drought than S. scoparium at both the population and organismal levels*

**Figure 1.**
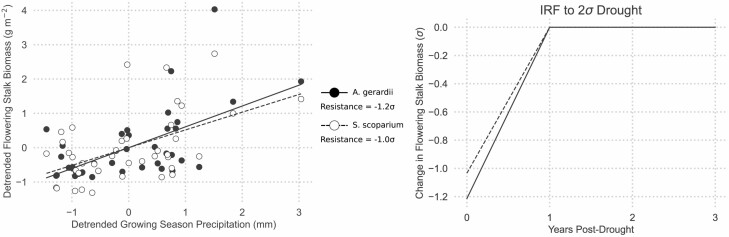
The relationship between precipitation and flowering stalk production was stronger for *A. gerardii* than for *S. scoparium*. This left panel shows linear relationship between detrended and standardized flowering stalk biomass and detrended, standardized growing season precipitation (April—September). The right panel shows the impulse response functions for *A. gerardii* and *S. scoparium* for three years following a 2*σ* drought in year 0. Resistance and IRFs were calculated following Lemoine (2021).

Long-term field observations supported our hypothesis that *A. gerardii* is more susceptible than *S. scoparium* to annual rainfall variability. Growing season precipitation was positively correlated with flowering stalk biomass for both grasses (Fig. 1), and neither *A. gerardii* nor *S. scoparium* exhibited temporal lag effects (see Supporting Information—Table S1). The lack of lag effects suggests that both species are capable of full recovery following a single year of drought. However, the instantaneous impact of drought on flowering stalk production differed between the species. *Schizachyrium scoparium* was modestly sensitive to a 2*σ* reduction in rainfall, incurring a 1*σ* reduction in flowering stalk biomass (Fig. 1). Given the stronger relationship between flower production and rainfall, *A. gerardii* lost approximately 20% more flowering stalk biomass than *S. scoparium* during the same standardized drought (Fig. 1).

**Figure 2.**
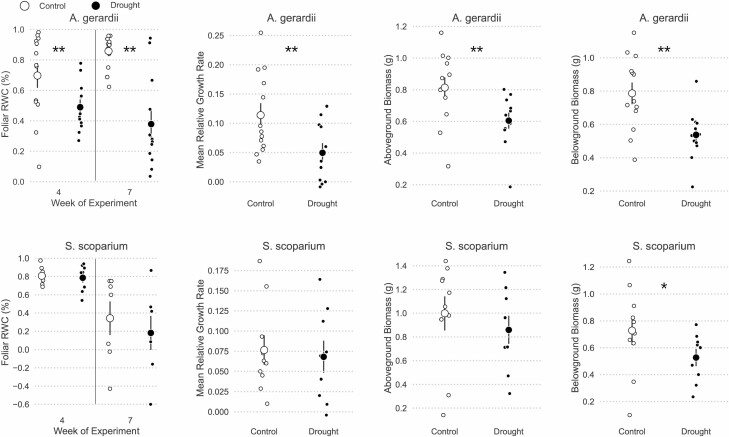
Organismal measures of *A. gerardii* were more drought-sensitive than for *S. scoparium*. This figure shows foliar RWC, maximum weekly height, end-of-experiment aboveground biomass production, and end-of-experiment belowground biomass production for both *A. gerardii* and *S. scoparium*. Small points show individual values, large points and bars show means ± 1 SE. Significant differences between watering treatments at Pr(0.95) are denoted with **, and moderate differences at Pr(0.90) are denoted with *.

Organismal measurements during the laboratory experiment confirmed that *S. scoparium* is more drought-tolerant than *A. gerardii*. First, *A. gerardii* experienced greater reductions in foliar RWC than *S. scoparium*. During the fourth week, water-limitation reduced *A. gerardii* foliar RWC by 21 ± 9% [Pr(drought < control | week 4) = 0.993, Fig. 2]. Deficits of foliar RWC were doubled in the seventh week [Pr(drought < control | week 7) > 0.999, Fig. 2]. In contrast, mean foliar RWC of *S. scoparium* was unaffected by drought at any time point [Pr(drought < control) < 0.85 for weeks 4 and 7, Fig. 2]. Likewise, low water availability reduced the mean relative growth rate of *A. gerardii* [Pr(drought < control) = 0.980] but not of *S. scoparium* [Pr(drought < control) = 0.609, Fig. 2]. Total aboveground biomass of *A. gerardii* was also reduced 0.126 ± 0.088 g by drought [Pr(drought < control) = 0.979], whereas drought had a negligible effect on mean aboveground biomass production of *S. scoparium* [Pr(drought < control) = 0.732, Fig. 2]. Likewise, drought significantly impaired *A. gerardii* belowground production [Pr(drought < control) = 0.996] and had only a moderate effect on belowground production of *S. scoparium* [Pr(drought < control) = 0.904, Fig. 2].

**Figure 3.**
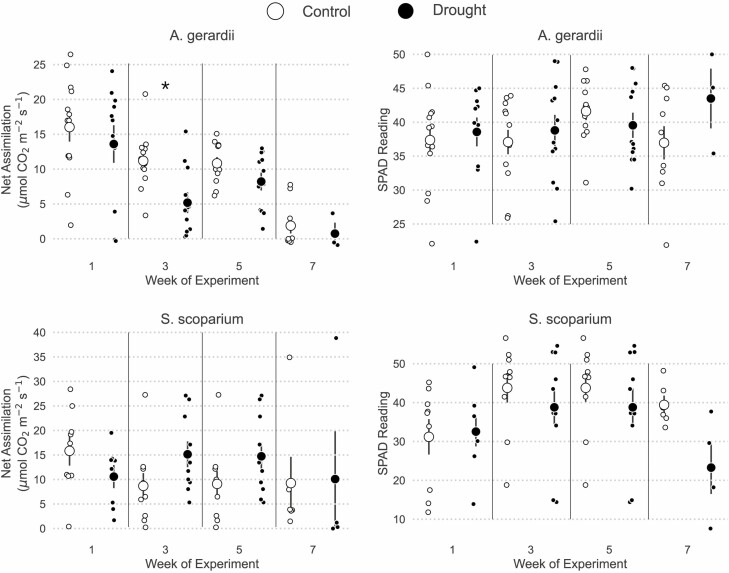
Physiological measures of *A. gerardii* performance were slightly more drought-sensitive than for S*. scoparium*. This figure shows net CO_2_ assimilation and SPAD chlorophyll readings over the course of the experiment for both *A. gerardii* and *S. scoparium*. Small points show individual values, large points and bars show means ± 1 SE. Significant differences between watering treatments at Pr(0.95) are denoted with **, and moderate differences at Pr(0.90) are denoted with *.

Physiologically, *A. gerardii* was slightly more drought sensitive than *S. scoparium*. Specifically, *A* of *A. gerardii* declined through time [Pr(< week 1) > 0.97 for all weeks, Fig. 3]. During weeks 1 and 5, the drought treatment had no effect on *A* [Pr(drought < control) < 0.80 for weeks 1,3 and 5, Fig. 3]. During the third week of the experiment, drought reduced mean *A* by approximately 50% [Pr(drought < ambient | week 3) = 0.943, Fig. 3). Unlike *A. gerardii*, photosynthesis of *S. scoparium* was unaffected by either the week of measurement or drought treatment [Pr(effect) < 0.6 for all week/treatment combinations, Fig. 3]. Leaf chlorophyll content, as estimated by the SPAD metre, was stable for both *A. gerardii* [Pr(effect) < 0.7 for nearly all week/treatment combinations] and *S. scoparium* [Pr(effect) < 0.8, Fig. 3]. The only exception was that *S. scoparium* showed a moderate increase in SPAD readings during week 5 (Pr(week 5 > week 1) = 0.901, Fig. 3].

*H2: Andropogon gerardii physiology was impacted by drought more than S. scoparium*

**Figure 4.**
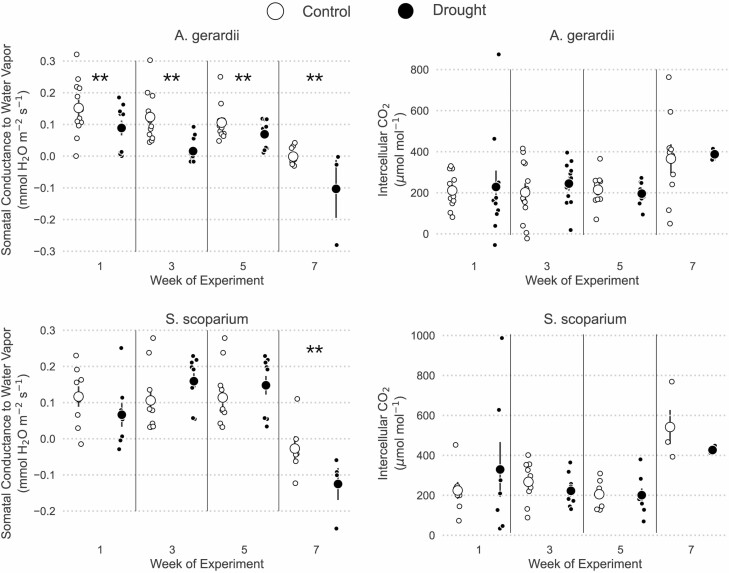
Measures of water use for *A. gerardii* were more drought-sensitive than for S*. scoparium*. This figure shows stomatal conductance to water vapor (*g*_sw_) and intercellular CO_2_ concentrations for both *A. gerardii* and *S. scoparium*. Small points show individual values, large points and bars show means ± 1 SE. Significant differences between watering treatments at Pr(0.95) are denoted with **, and moderate differences at Pr(0.90) are denoted with *.

Contrary to our expectations, *S. scoparium* did not avoid drought by reducing water loss through stomata, although the two species differed substantially in how water stress affected *g*_sw_. Stomatal conductance for *A. gerardii* was similar between weeks 1 and 3 [Pr(week 1 < week 3) = 0.804], before declining in weeks 5 and 7 [Pr(weeks 5,7 < week 1) > 0.922, Fig. 4]. Overall, drought reduced *g*_sw_ of *A. gerardii* in all weeks [Pr(drought < control) = 0.993], though this effect was moderately more severe during the third week than in other weeks [Pr(week 3 interaction) = 0.941, Fig. 4]. In contrast to *A. gerardii*, *S. scoparium* maintained stomatal function during drought across weeks 1, 3 and 5 [Pr(drought < control) < 0.782 for weeks 1, 3 and 5; Fig. 4]. Only during week 7 did drought significantly reduce *g*_sw_ of *S. scoparium* [Pr(drought < control | week 7) = 0.976] (Fig. 4). Though the data appear to show that *g*_sw_ was higher under drought during weeks 3 and 5 (Fig. 4), this effect was not significant for either week 3 [Pr(drought > control | week 3) = 0.884] or week 5 [Pr(drought > control | week 5) = 0.785]. Also note that some *g*_sw_ were below 0. These negative values all occurred in extremely dry plants (RWC < 30%) and indicate that stomatal conductance was either too low to measure or that the dry leaves, when placed in the humid leaf chamber, did actually uptake water.

Despite the reduction in stomatal function during drought, *A. gerardii* maintained constant *Ci* both during drought [(Pr(drought < control) = 0.473] and across time [Pr(effect) < 0.70 for weeks 3 and 5, Fig. 4]. During week 7, *Ci* of *A. gerardii* did increase significantly [Pr(week 7 > week 1) = 0.972, Fig. 4]. Patterns in *Ci* were similar for *S. scoparium.* Drought had no effect on *Ci* in any week, but *Ci* was significantly higher in week 7 than in all other weeks [Pr(week 7 > week 1) = 0.990, Fig. 4].

**Figure 5.**
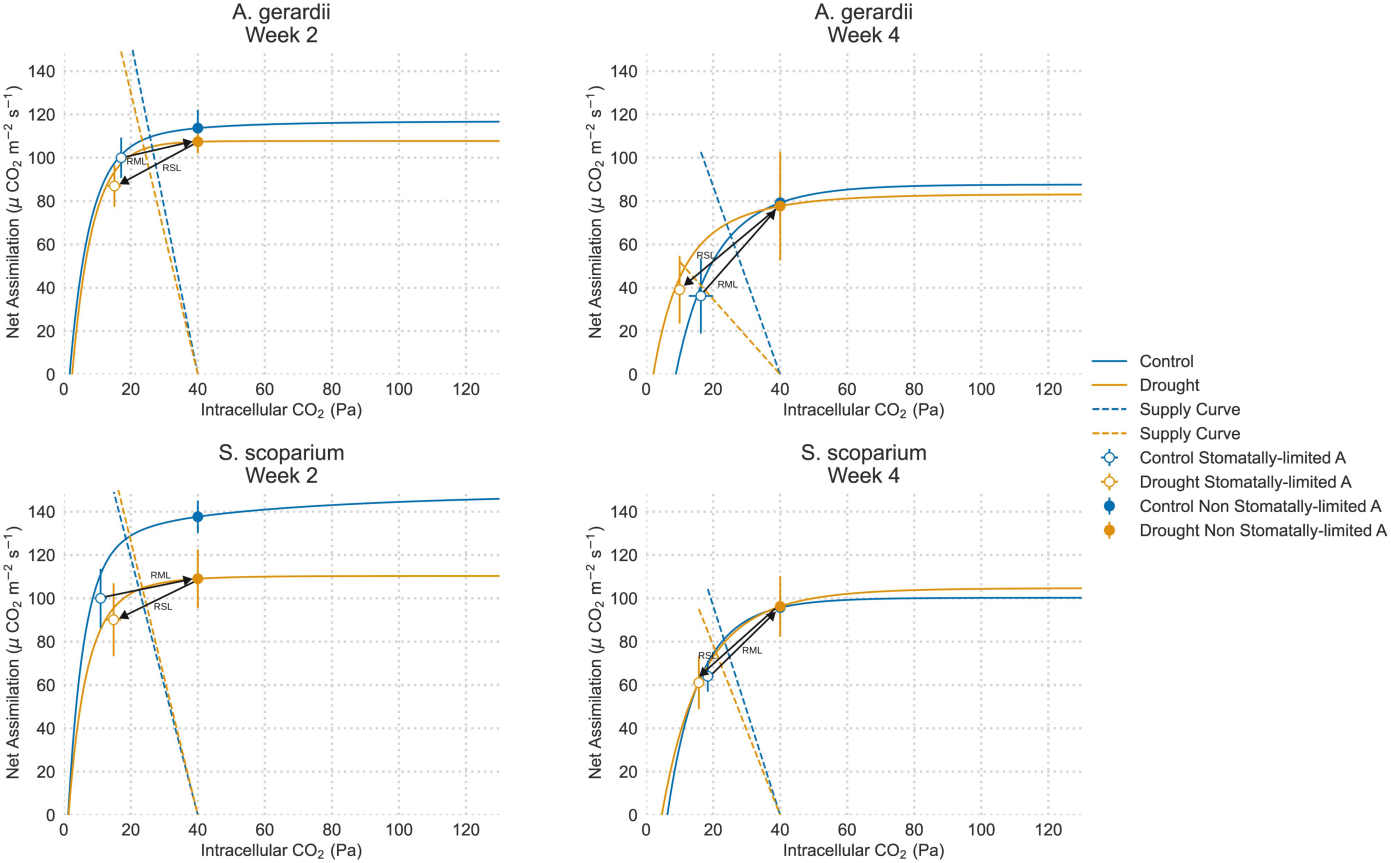
*A*-*Ci* curves for *A. gerardii* and *S. scoparium* in control and drought conditions. Photosynthetic rates are expressed as a percentage of the observed A under stomatal limitation during week 2 (X, during week 2). Lines show the average *A-Ci* curve for each treatment. Panels show *A-Ci* curves for (A) *A. gerardii* during week 2, (B) *A. gerardii* during week 4, (C) *S. scoparium* during week 2, and (D) *S. scoparium* during week 4.

Reduced stomatal conductance during later drought stages had the effect of substantially lowering CO_2_ supply relative to demand for *A. gerardii* (Fig. 5). In week 2, *A. gerardii* maintained relatively constant CO_2_ supply and demand under drought (Fig. 5A). However, by the fourth week of the experiment, CO2 supply was greatly reduced in *A. gerardii*, suggesting that CO_2_ limitation became a prevailing factor in limiting photosynthesis (Fig. 5B). In contrast, CO_2_ supply relative to demand was generally unaffected by drought for *S. scoparium* during both weeks 2 and 4 of the drought treatment (Figs. 5C-D).

*H3: Relative stomatal and metabolic limitation under drought did not differ between A. gerardii and S. scoparium*

**Figure 6.**
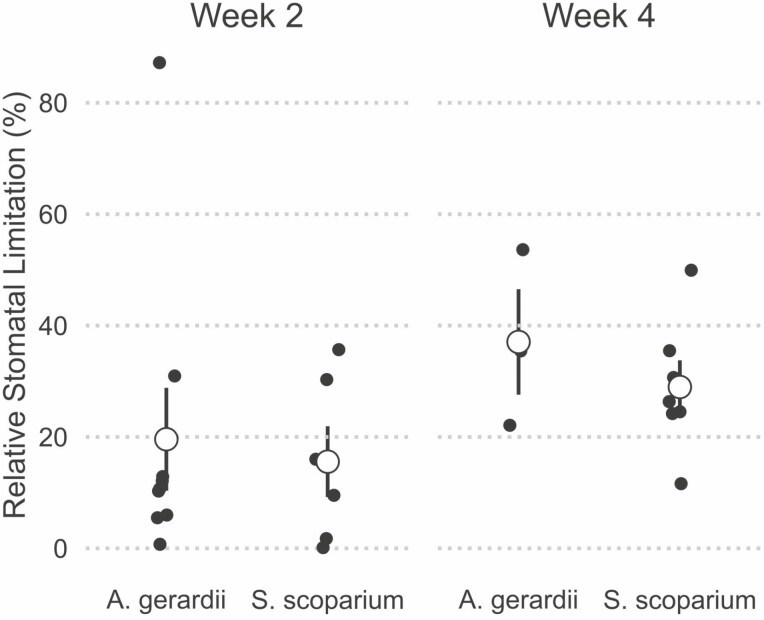
Relative stomatal limitation did not differ between species. This figure shows relative stomatal limitation for both *A. gerardii* and *S. scoparium*. Small points show individual values, large points and bars show means ± 1 SE. Significant differences between species at Pr(0.95) are denoted with **, and moderate differences at Pr(0.90) are denoted with *.

**Figure 7.**
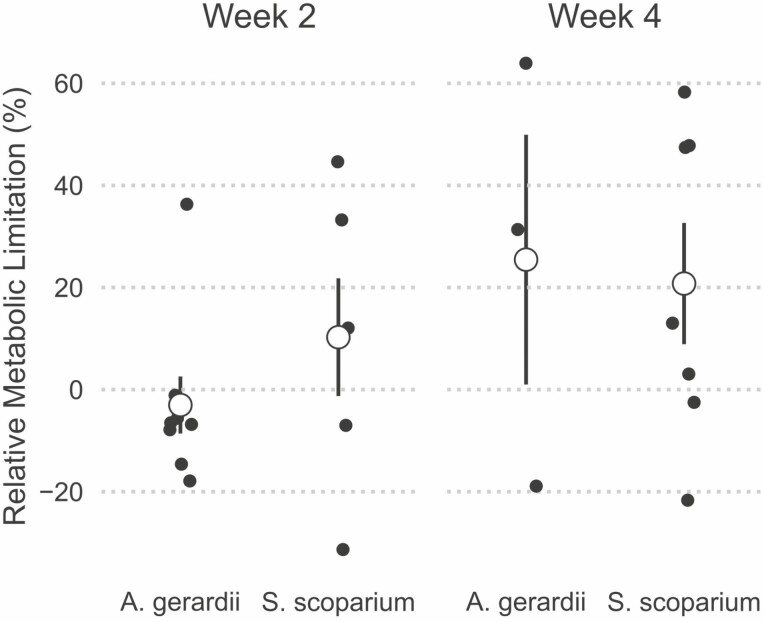
Relative metabolic limitation did not differ between species. This figure shows the relative metabolic limitation for both *A. gerardii* and *S. scoparium*. Small points show individual values, large points and bars show means ± 1 SE. Significant differences between species at Pr(0.95) are denoted with **, and moderate differences at Pr(0.90) are denoted with *.

We expected that *S. scoparium* would exhibit stronger stomatal limitation of photosynthesis under drought, while drought would impose severe metabolic limitations on photosynthesis by *A. gerardii*. Relative stomatal limitation did not differ either by species [Pr(*S. scoparium *> *A. gerardii) *= 0.658), by week [Pr(week 4 > week 2) = 0.845], or by the interaction between species and week [Pr(interaction) = 0.549, Fig. 6]. Relative metabolic limitation was highly variable among individuals, but did not vary systematically among species [Pr(*S. scoparium *> *A. gerardii) *= 0.699), between weeks [Pr(week 4 > week 2) = 0.846], or with the interaction between species and week [Pr(interaction) = 0.533, Fig. 7]. Negative RML values can be explained by examining the supply-demand curves, and occur when *A*_40-drought_ was greater than *A*_obs-control_.

## Discussion

The increased frequency of droughts poses a severe threat to grassland stability and function. Understanding why dominant, co-occurring species of the same functional type exhibit different drought responses will enable us to predict how grasslands will be affected by future water shortages. Although the different drought responses of *A. gerardii* and *S. scoparium* have been well-documented, few studies provide a detailed physiological examination underlying why these two species differ. Based on the available data, we predicted that (H1) *S. scoparium* would be more drought resistant than *A. gerardii*, (H2) *S. scoparium* would exhibit greater stomatal restriction than *A. gerardii* under drought, and (H3) *A. gerardii* would be more metabolically limited under drought than *A. gerardii*. Our results supported the prediction that *S. scoparium* is more drought-resistant than *A. gerardii* based on long-term field data, organismal biomass production and physiological gas exchange measurements. In particular, *S. scoparium* maintained high CO_2_ supply rates under drought in contrast to *A. gerardii*, that enabled it to avoid photosynthetic limitation. That two common, co-occurring C_4_ species possess such different responses to drought highlights the physiological variability inherent within plant functional groups and underscores the need for more studies of C_4_ drought tolerance.

In natural settings, *S. scoparium* is generally less affected by interannual variation in rainfall than *A. gerardii.* During drought, *A* of *A. gerardii* declines more severely than that of *S. scoparium*, which is often not affected by drought (Maricle and Adler 2011; Lemoine and Budny 2022). However, lower photosynthetic rates of *A. gerardii* do not always translate into reduced biomass production in field experiments (Fay *et al.* 2003; Hoover *et al.* 2014a; b). There are many potential reasons why field experimental results might differ from pot experiments, including a general tendency for potted soils to drain rapidly (Ray and Sinclair 1998) and for potted plants to have underdeveloped root systems (Poorter *et al.* 2012). In the field, *A. gerardii* roots can extend over 1 m deep, and it is possible that a robust root system enables plants to maintain production during drought despite reduced photosynthetic capacity by transporting carbon and nitrogen from roots to shoots. In fact, most direct comparisons of drought tolerance between *S. scoparium* and *A. gerardii* were conducted on potted plants (Heckathorn and Delucia 1994, 1996; Heckathorn *et al.* 1997). Field experiments supported the conclusion that *S. scoparium* is less drought sensitive than *A. gerardii*, but with smaller differences between the two species (Maricle and Adler 2011; Maricle *et al.* 2015). Our study, in conjunction with others, suggests that the differences between these two species’ drought tolerance in both pot and field conditions are likely determined by stomatal responses and maintenance of water status.

Non-stomatal limitations occur when the rates of either Rubisco carboxylase activity (*V*_cmax_) or electron transport for RuBP regeneration (*J*_max_) limit photosynthesis. In many C_3_ grass species, both *V*_cmax_ and *J*_max_ can inhibit CO_2_ fixation during drought (Signarbieux and Feller 2011; Reed and Loik 2016). Compared to C_3_ plants, however, leaf biochemistry of C_4_ species has been understudied. Common garden and pot experiments suggest that C_4_ photosynthesis is metabolically limited during drought (Ripley *et al.* 2007, 2010; Taylor *et al.* 2014), which is to be expected given that the C_4_ pathway is saturated at lower *Ci* to avoid stomatal limitation. Though previous work has suggested that metabolic limitations are prevalent for *A. gerardii* and *S. scoparium* (Maricle and Adler 2011; Maricle *et al.* 2015), no study has yet directly measured RSL and RML. Our study suggests that RSL, averaging 20–40%, was greater than RM, which averaged 0–20%. Yet despite the similar RSL for both species, only *A. gerardii* showed a decrease in CO_2_ supply as a result of lower stomatal conductance.

Notwithstanding the similarity in RSL and RML for both species, *S. scoparium* and *A. gerardii* showed quite different photosynthetic responses to drought. Water limitation did not affect *g*_sw_ of *S. scoparium*, which enabled it to maintain high *Ci* and therefore high *A* even under drought conditions. Alternatively, drought did cause stomatal closure of *A. gerardii*, which was able to maintain high *Ci* despite reduced stomatal conductance. Our SPAD measurements suggest that drought did not affect chlorophyll content of *A. gerardii* and instead must have reduced the efficiency by which photosynthesis occurred via some metabolic process not accounted for by our measurements of RML. There are many possible explanations that cannot be teased apart from our measurements. Importantly, *Ci* measures only the intercellular CO_2_ concentration and does not measure CO_2_ within the mesophyll or bundle sheath cells where the C_3_ and C_4_ pathways occur, respectively. Mesophyll (*g*_m_) and bundle sheath (*g*_bs_) conductance to CO_2_ have generally been assumed to be high, and our methods of RML assume that both *g*_m_ and *g*_bs_ are unlimited. However, recent studies have shown that *g*_m_ and *g*_bs_ can vary by 300% among C_4_ species, and that combined these two processes can account for over a third of photosynthetic limitation (Cano *et al.* 2019). It seems likely that *A. gerardii* might be CO_2_ limited by internal CO_2_ diffusion at *g*_m_ or *g*_bs_, despite maintaining high *Ci*. Internal diffusion limitation would match our results showing reduced CO_2_ supply of *A. gerardii* limits photosynthesis under drought. Yet how these diffusion constraints are affected by drought remains unknown.

Our study also supports the hypothesis that tradeoffs exist between drought strategies. Plant hydraulic traits often show tradeoffs between phenotypes that confer drought-tolerance and those that confer drought-avoidance (Ramírez-Valiente and Cavender-Bares 2017; Fu and Meinzer 2018; Bartlett *et al.* 2019). In grasses, trade-offs occur between ‘boom-and-bust’ species, with fast-growing productive species being the most drought intolerant (Griffin-Nolan *et al.* 2023). Here, we demonstrate that the degree of stomatal limitation also trades-off with the degree of metabolic limitation. It is possible that these strategies reflect the isohydric/anisohydric continuum, with stomatal limitation being more important for anisohydric plants that vary internal water content, and metabolic limitations being more important for isohydric plants that are able to maintain high internal water pressures but reduce growth (Fu and Meinzer 2018). Alternatively, isohydry appears to be a common strategy for drought-tolerant plants from arid sites, and anisohydry is more common for plants from wet habitats (Jardine *et al.* 2021). In our study, *S. scoparium* appeared to be more anisohydric and also more drought-tolerant, while *A. gerardii* is isohydric but experiences severe metabolic limitation under drought. Thus, it is possible that the tradeoff between metabolic and stomatal limitation reflects a more broad life history tradeoff.

Droughts are increasing in prevalence throughout the world. Predicting the impacts of drought on ecosystem function is therefore a primary goal of grassland ecologists (Wilcox *et al.* 2020). However, most ecosystem models oversimplify a system by treating all grass species within a single functional type as homogenous. Here, our results support previous studies in showing that co-occurring species of a single functional type can exhibit widely divergent metabolic, physiological and organismal responses to drought. Understanding how species vary in these traits could provide critical information needed to understand how grasslands will be impacted by global change in the future.

## Supporting Information

The following additional information is available in the online version of this article –

**Table S1.** Model selection criteria for AR(0), AR(1) and AR(2) models for *A. gerardii* and *S. scoparium.*

**Figure S1.** Graphical depiction of an IRF. In this IRF, the ecosystem is subject to an initial shock in the exogenous predictor with magnitude *α*. The system then returns to its average conditions for the rest of the time series. Resistance is measured by the decline in ecosystem state during the shock, with more negative values implying less resistance. Recovery is the extent to which an ecosystem remains altered post-disturbance, with more negative values implying less recovery. Elasticity is the rate at which the system recovers (i.e. slope, Δ*y*/Δ*x*), and return time is the amount of time it takes for a system to return to nominal levels. Note that this example implies a harmful disturbance. However, the sign of all values and the curve could flip for positive disturbances, such as a pulse of nitrogen enrichment on plant production. Taken from Lemoine (2021).

**Figure S2.** Comparison of steady-state (SS) and dynamic assimilation (DA) *A*-*Ci* curves.

plae012_suppl_Supplementary_Tables_S1_Figures_S1-S2

## Data Availability

All raw data, cleaned data, Python scripts and figures are available on Figshare at 10.6084/m9.figshare.24319147.
