# Supplementary material for: Schizachyrium scoparium (C4) better tolerates drought than Andropogon gerardii (C4) via constant CO2 supply for photosynthesis during water stress
Source: AoB Plants. 2024 Mar 8;16(2):plae012. doi: 10.1093/aobpla/plae012 (PMC10944017; doi:10.1093/aobpla/plae012)

Table S1. Model selection criteria for AR(0), AR(1), and AR(2) models for *A. gerardii* and *S. scoparium.*

Andropogon gerardii

Model	BIC	dBIC
**AR(0)**	**101.5**	**0.0**
AR(1)	105.2	3.7
AR(2)	108.9	7.4

Schizachyrium scoparium

Model	BIC	dBIC
**AR(0)**	**107.4**	**0.0**
AR(1)	110.0	2.6
AR(2)	113.7	6.3

Figure S1. Graphical depiction of an IRF. In this IRF, the ecosystem is subject to an initial shock in the exogenous predictor with magnitude α. The system then returns to its average conditions for the rest of the time series. Resistance is measured by the decline in ecosystem state during the shock, with more negative values implying less resistance. Recovery is the extent to which an ecosystem remains altered post-disturbance, with more negative values implying less recovery. Elasticity is the rate at which the system recovers (i.e. slope, Δy/Δx), and return time is the amount of time it takes for a system to return to nominal levels. Note that this example implies a harmful disturbance. However, the sign of all values and the curve could flip for positive disturbances, such as a pulse of nitrogen enrichment on plant production. Taken from Lemoine (2021).

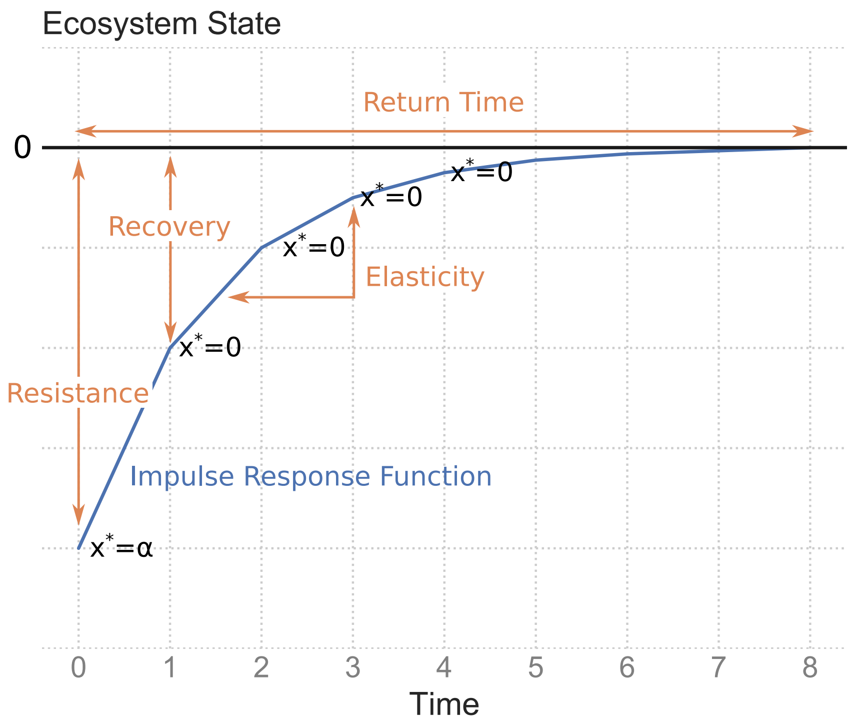

Figure S2. Comparison of steady-state (SS) and dynamic assimilation (DA) A-Ci curves.

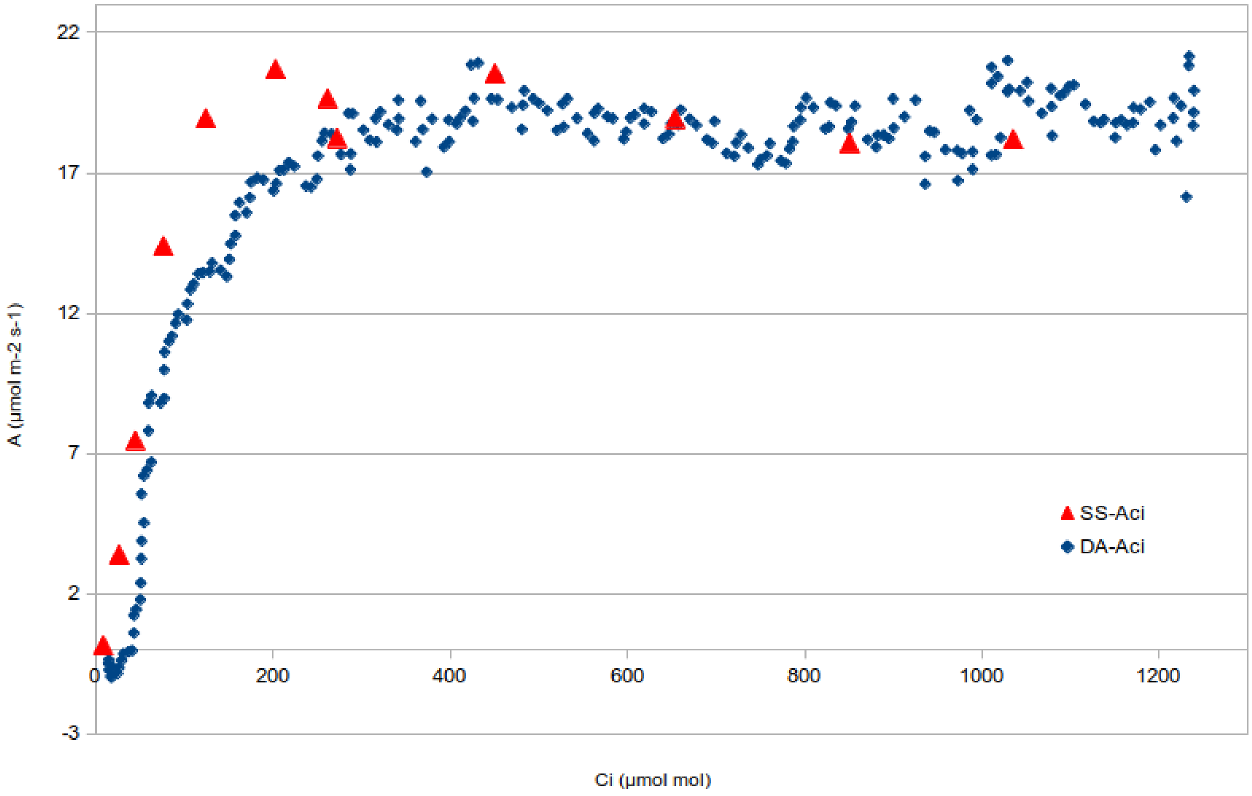

Supplement: plae012_suppl_Supplementary_Tables_S1_Figures_S1-S2 [file plae012_suppl_supplementary_tables_s1_figures_s1-s2.pdf]
